# General health condition of patients hospitalized after an incident of in‐hospital or out‐of hospital sudden cardiac arrest with return of spontaneous circulation

**DOI:** 10.1002/clc.23680

**Published:** 2021-07-27

**Authors:** Michał Stasiowski, Łukasz Głowacki, Jakub Gąsiorek, Dominika Majer, Ewa Niewiadomska, Seweryn Król, Jakub Żak, Anna Missir, Lech Krawczyk Prof, Przemysław Jałowiecki Prof, Beniamin Oskar Grabarek

**Affiliations:** ^1^ Department of Anaesthesiology and Intensive Therapy Regional Hospital Sosnowiec Poland; ^2^ Department of Emergency Medicine, School of Medical Sciences in Zabrze Medical University of Silesia Katowice Poland; ^3^ Students Scientific Society by Department of Emergency Medicine, School of Medical Sciences in Zabrze Medical University of Silesia Katowice Poland; ^4^ Department of Epidemiology and Biostatistics, School of Public Health in Bytom Medical University of Silesia Katowice Poland; ^5^ Department of Histology, Cytophysiology, and Embryology, Faculty of Medicine in Zabrze The University of Technology in Katowice Katowice Poland

**Keywords:** Glasgow outcome scale, intensive care unit, lactate concentration, return of spontaneous circulation, sudden cardiac arrest

## Abstract

**Background:**

Sudden cardiac arrest (SCA) is one of the main reasons for admission to the intensive care unit (ICU), which influences discharge in a good neurological state.

**Hypothesis:**

To analyze patients who had recovery of spontaneous circulation (ROSC) during hospitalization in the ICU using the Glasgow Outcome Scale (GOS).

**Methods:**

The study group comprised 78 patients after SCA (35 after out‐of‐hospital cardiac arrest [OHCA] and 43 after in‐hospital cardiac arrest [IHCA]) with ROSC who were admitted to the ICU of Regional Hospital No. 5 in Sosnowiec from January 1, 2016 to December 31, 2016. GOS was used to assess neurological status. Basic anthropological data, with, arterial blood pH, lactate concentration (LAC), and catecholamine treatment were also collected.

**Results:**

In the study group, 32.1% (*n* = 25/78) of patients survived until ICU discharge and 30.8% (*n* = 24/78) until discharge from the hospital. SCA in cardiac mechanism was more common in OHCA than in the IHCA group (OHCA vs. IHCA: 85.7% vs. 62.8%, *p* = .02). There was no statistically significant difference between the two groups for neurological status assessed using GOS. There was no statistically significant difference between LAC or arterial blood pH and survival to ICU discharge, survival to hospital discharge, or mortality. The need for using catecholamines increased the mortality rate (GOS 1) (*p* < .001).

**Conclusions:**

Most patients after RSOC were assigned to a group other than GOS 1, and 25% of all subjects belonged to GOS 4–5. Treatment with catecholamines was more common in patients who do not survive hospital or ICU discharge.

## 1 INTRODUCTION

Sudden cardiac arrest (SCA) is one of the main reasons for admission to the intensive care unit (ICU), and it is defined as a sudden loss of mechanical heart activity.^1^ Mortality in industrialized countries due to SCA is approximately 20%.^2^ SCA is most common in patients with cardiological burden.^3^ In the United States, approximately 395 000 cases of out‐of‐hospital cardiac arrest (OHCA) and approximately 200 000 cases of in‐hospital cardiac arrest (IHCA) are found every year. According to the European Resuscitation Council, the rate of incidence of SCA fluctuates approximately from 350 000 to 700 000 in Europe every year.^4^

In 2018, the Polish state emergency medical system reported 26 738 cardiopulmonary resuscitation (CPR) attempts in OHCA (analysis of OHCA in Poland in a 12‐month period).

In recent years, the rate of return of spontaneous circulation (ROSC) has increased because of improvement in the quality of medical services. After CPR, it is not only important to obtain ROSC and reduce mortality, but also survival to hospital discharge in a good neurological condition.^6^

Several studies have shown that even the shortest delay in performing chest compressions can significantly reduce the chance of ROSC as well as survival in a good neurological condition.^4^, ^7^, ^8^ Improving the quality of CPR was possible due to the latest advancements in medical technologies. The use of devices for automatic chest compressions or real‐time monitoring of quality chest compressions is becoming an increasingly common method for improving CPR. It is used mainly to transport patients during CPR after SCA to a hospital as well as allow the performance of medical procedures, such as coronarography.^9^, ^10^ Additionally, the prediction of ROSC and neurological outcome after CPR depends on factors such as the time of CPR, total dosage of adrenaline during CPR, and the first electrocardiographic rhythm (ECG).^11^ Ventricular fibrillation (VF) and pulseless ventricular tachycardia (pVT) are very often the first ECG rhythms recorded in patients who suffered SCA. Defibrillation rhythms have been found to correlate positively with the increased possibility of ROSC compared with non‐defibrillation rhythms (pulseless electrical activity and asystole).^12^, ^13^ In the case of shock‐refractory pVT and VF, the use of amiodarone or lidocaine is recommended. Amiodarone inhibits the potassium channel, which prolongs the duration of the third phase of the action potential of conducting cells in the heart. In turn, lidocaine inhibits the sodium permeability of the neuron's membrane by blocking the sodium‐potassium pump. These drugs have a similar effect on survival during CPR with shock‐resistant pVT and VF.^14^

During hypoxia, the cells of the cerebral cortex, which are the most sensitive to oxygen deficiency, begin to die by necrosis and apoptosis. Longer durations of hypoxia cause more brain damage^15^; however, there are known methods to reduce brain damage after SCA, such as hypothermia or extracorporeal membrane oxygenation (ECMO). Targeted temperature management can lead not only to hospital discharge survival but also to discharge from the hospital in good neurological condition. Currently, several methods of inducing hypothermia after SCA are known, such as cold fluid infusion, external cooling, peritoneal lavage, and intravascular cooling. Nevertheless, the abovementioned medical techniques are very rare due to medical complications and their limited availability.^16^, ^17^, ^18^, ^19^

Utstein guidelines have been implemented for easily comparing CPR data of different countries and medical centers. They enable a uniform method of SCA data presentation.^20^, ^21^ According to American Heart Association report, the rate of survival to hospital discharge after OHCA among the adult population of the United States in the year 2015 fluctuated from 10.6% to 12.4%, whereas the survival rate after IHCA remained at 26.4% in 2016.^22^, ^23^ The data revealed by the European Resuscitation Council on the survival rate in Europe in the case of OHCA amounted to an average of 6.4% and in the case of IHCA between 3% and 27%. In Poland, survival depending on the time of contact with a healthcare professional in the hospital was approximately 36.3%.^4^, ^5^, ^24^, ^25^, ^26^

The Glasgow Outcome Scale (GOS), which was first used to assess neurological dysfunction and quality of life (QoL) after head injury^27^ because of its simplistic use and is gaining more popularity for assessing the QoL after SCA with ROSC.^28^

## 2 METHODS

All patients after SCA (either OHCA or IHCA) and successful resuscitation defined as ROSC before admission to the ICU of Regional Hospital No. 5 in Sosnowiec from January 1, 2016 to December 31, 2016 were included in the study.

The patient's health condition upon discharge from the ICU was assessed using the GOS. Furthermore, we analyzed additional factors that determine the prognosis after SCA with ROSC, such as arterial blood pH, lactate concentration (LAC) on admission to the ICU, and treatment with catecholamine during therapy. We also analyzed the time of treatment in the ICU, patient survival to discharge from ICU, time of treatment between discharge from ICU to discharge from the hospital, and patient survival to discharge from the hospital.

Statistical analysis was performed using MS Excel and Statistica 12.5, StatSoft Poland, R 3.1.2, GNU GPL.

The measurable data are presented as mean and standard deviation (SD) or median and quantiles [M (Q1 Q3)] depending on distribution, which agreed with normal distribution and was measured using the Shapiro–Wilk test.

The analysis of the significant difference in the mean value between groups concerning the type cardiac arrest (OHCA, IHCA) was performed using Student's *t*‐test or Mann–Whitney *U* test, whereas, in GOP, the pH and LAC groups were subjected to one‐way analysis of variance or the Kruskal–Wallis test.

The qualitative data are presented as percentages, and results for dependent variables were obtained using the χ ^2^ test, whereas comparisons among groups were performed using the Difference Fraction Significance test with Bonferroni correction for multiple comparisons.

*p*‐value <.05 was considered statistically significant. Informed consent was obtained from all of the patients recruited.

## 3 RESULTS

Seventy‐nine patients who suffered SCA with ROSC were admitted to the ICU; one patient was excluded from the final analysis due to the sudden death after admission before any gasometric blood samples were taken. Therefore, 78 patients after an episode of SCA with ROSC who were admitted to the ICU of Regional Hospital No. 5 in Sosnowiec from January 2016 to December 2016 were included in the final statistical analysis. The patients were divided into two groups according to the setting of SCA: OHCA‐35 patients (44.9%) and IHCA‐43 patients (55.1%). No statistically significant differences in anthropometric data were recorded between the two analyzed groups of patients (Table 1).

**TABLE 1:** Anthropometric data of patients admitted to the ICU after SCA with ROSC

Anthropometric data	Total	OHCA group	IHCA group	*p*‐value
	*n* = 78 (100%)	*n* = 35 (44.9%)	n = 43 (55.1%)	
Age, years mean (SD)	62.2 (15.7)	60.8 (17.3)	63.3 (14.3)	.48 NS
Age < 40 years *n* (%)	7 (9)	4 (11.4)	3 (7)	.5 NS
Age 40–65 years *n* (%)	31 (39.7)	14 (40)	17 (39.5)	.96 NS
Age > 65 years *n* (%)	40 (51.3)	17 (48.6)	23 (53.5)	.67 NS
Male/female *n* (%)	48 (61.5)/30 (38.5)	22 (62.9)/13 (37.1)	26 (60.5)/17 (39.5)	.83 NS

Abbreviations: ICU, intensive care unit; IHCA, in‐hospital cardiac arrest; NS, not significant; OHCA, out‐of‐hospital cardiac arrest; ROSC, return of spontaneous circulation; SCA, sudden cardiac arrest.

A higher proportion of patients after OHCA in cardiac mechanism vs. the proportion of patients after IHCA (85.7% vs. 62.8%), as well as a lower proportion of patients after OHCA in non‐cardiac mechanism vs. the proportion of patients after IHCA (13.3% vs. 37.2%), were observed; both above‐mentioned differences were statistically significant (*p* < .05). Between the groups analyzed (IHCA vs. OHCA), no statistically significant differences were observed for the survival rate to hospital discharge, duration of hospitalization after ICU discharge, or duration of hospitalization in the ICU. Similarly, neither the necessity of catecholamines nor the level of pH and LAC on admission to the ICU appeared to be statistically significant (Table 2).

**TABLE 2:** Selected variables of patients after OHCA and IHCA with ROSC

SCA‐treatment	TOTAL	OHCA	IHCA	*p*‐value
	*n* = 78 (100%)	*n* = 35 (44.9%)	*n* = 43 (55.1%)	
SCA in cardiac mechanism (%)	57 (73.1)	30 (85.7)	27 (62.8)	.02 *p* < .05
SCA in non‐cardiac mechanism (%)	21 (26.9)	5 (14.3)	16 (37.2)	.02 *p* < .05
Unknown cause of SCA *n* (%)	6 (7.7)	1 (2.9)	5 (11.6)	.15 NS
pH on admission to the ICU *M* (Q1÷Q3)	7.4 (7.22÷7.46)	7.41 (7.22÷7.46)	7.39 (7.24÷7.46)	.69 NS
LAC on admission to the ICU *M* (Q1÷Q3)	2.75 (1.55÷6.85)	2.45 (1.6÷6)	2.95 (1.5÷9.8)	.58 NS
Catecholamine treatment in ICU *n* (%)	60 (78.9)	24 (72.7)	36 (83.7)	.24 NS
Survival to ICU discharge *n* (%)	25 (32.1)	13 (37.1)	12 (27.9)	.38 NS
Time of hospitalization in the ICU, days *M* (Q1÷Q3)	7 (3÷17)	8 (4÷21)	6 (2÷12)	.12 NS
Time of hospitalization after ICU discharge, days *M* (Q1÷Q3)	6 (3÷21)	6 (3÷27)	8 (2.5÷21)	.87 NS
Survival to hospital discharge *n* (%)	24 (30.8)	12 (34.3)	12 (27.9)	.54 NS

Abbreviations: ICU, intensive care unit; IHCA, in‐hospital cardiac arrest; LAC, lactate concentration; M (Q1÷Q3), median and quantiles; NS, not significant; OHCA, out‐of‐hospital cardiac arrest; ROSC, return of spontaneous circulation; SCA, sudden cardiac arrest.

Dependence between the health condition, which was measured by the GOS scale and mechanism and place of cardiac arrest, pH value, and LAC on admission to the ICU, as well as the administration of catecholamines during the treatment, time of hospitalization in the ICU and after discharge from the ICU, and survival to discharge from the hospital were analyzed in detail.

Patients with good neurological status, defined as GOS scale 4–5, accounted for 24.4% (*n* = 19) (OHCA 28.6% [*n* = 10] and IHCA 20.9% [*n* = 9], respectively).

Patients with poor neurological status (GOS 2–3) accounted for 7.7% (*n* = 6) in the research group and deceased patients (GOS 1) 67.9% (*n* = 53).

The need for treatment with catecholamines during hospitalization in the ICU was indeed more frequent among the group of deceased patients 49 (94.2%) in contrast to other groups 2 (33.3%) for GOS 2–3 and 9 (50%) for GOS 4–5, respectively.

Surprisingly, the patient's sex significantly influenced their health condition. The health condition of males was better than that of females.

The proportion of deaths was comparable in both (Table 3).

**TABLE 3:** Relationship between the health condition of patients evaluated using GOS and selected variables of patients after SCA with ROSC

Glasgow Outcome Scale (GOS)	GOS 1	GOS 2–3	GOS 4–5	*p*‐value
	*n* = 53 (67.9%)	*n* = 6 (7.7%)	*n* = 19 (24.4%)	
IHCA *n* (%)	31 (58.5)	3 (50)	9 (47.4)	.68 NS
OHCA *n* (%)	22 (41.5)	3 (50)	10 (52.6)	.68 NS
SCA in cardiac mechanism *n* (%)	39 (73.6)	3 (50)	15 (78.9)	.41 NS
SCA in non‐cardiac mechanism *n* (%)	14 (26.4)	3 (50)	4 (21.1)	.41 NS
Unknown mechanism of SCA *n* (%)	3 (5.7)	1 (16.7)	2 (10.5)	.56 NS
pH on admission to the ICU M (Q1÷Q3)	7.4 (7.24÷7.46)	7,44 (7.22÷7.46)	7.41 (7.22÷7.46)	.91 NS
LAC on admission to the ICU, mM M (Q1÷Q3)	2.9 (1.6÷10)	3.2 (1.6÷5.5)	2.5 (1÷5.8)	.52 NS
Catecholamine treatment in ICU *n* (%)	49 (94.2)	2 (33.3)	9 (50)	*p* < .0001 GOS 1 vs. GOS 2–3*** GOS 1 vs. GOS 4–5***
Time of hospitalization in the ICU, days M (Q1÷Q3)	6 (2÷12)	34 (7÷52)	7 (3÷17)	.07 NS
Time of hospitalization after ICU discharge, days M (Q1÷Q3)	—	3 (0÷6)	8 (4÷27)	.21 NS
Survival to hospital discharge *n* (%)	—	5 (83.3)	19 (100)	.07 NS
Male/female *n* (%)	30 (56.6)/23 (43.4)	2 (33.3)/4 (66.7)	16 (84.2)/3 (15.8)	.03 *p* < .05

Abbreviations: ICU, intensive care unit; IHCA, in‐hospital cardiac arrest; LAC, lactate concentration; M (Q1÷Q3), median and quantiles; NS, not significant; OHCA, out‐of‐hospital cardiac arrest; ROSC, return of spontaneous circulation; SCA, sudden cardiac arrest.

*** *p* < .0001.

Additional analysis was also performed for survival rate and the duration of hospitalization (Table 4).

**TABLE 4:** Analysis of the relationship between arterial blood pH, lactate concentration and catecholamine treatment, and survival and treatment time in individual hospital areas

	pH			*p*‐value	LAC, mM			*p*‐value	Catecholamine treatment		*p*‐value
Parameter Reference range	6.7–7.3	7.3–7.5	>7.5		0–4	4–8	>8		Yes	No	
	*n* = 27 (34.6%)	*n* = 40 (51.3%)	*n* = 9 (11.5%)		*n* = 45 (57.7%)	*n* = 14 (17.9%)	*n* = 17 (21.8%)		*n* = 60 (76.9%)	*n* = 16 (20.5%)	
Survival to ICU discharge *n* (%)	9 (33.3)	14 (35)	2 (22.2)	.75 NS	16 (35.6)	6 (42.9)	3 (17.6)	.25 NS	11 (18.3)	13 (81.3)	*p* < .0001
Time of hospitalization in the ICU, days M (Q1÷Q3)	5 (1÷17)	7 (3÷16.5)	12 (6÷23)	.11 NS	7 (4÷19)	8.5 (6÷21)	2 (1÷6)	.02 NS	6.5 (2÷12)	7 (3÷18)	.47 NS
Time of hospitalization after ICU discharge, days M (Q1÷Q3)	11 (7÷34)	5 (0÷21)	3 (0÷6)	.09 NS	4.5 (0÷18.5)	16 (7÷21)	8 (5÷81)	.11 NS	7 (5÷39)	5 (0÷11)	.25 NS
Survival to hospital discharge *n* (%)	8 (29.6)	14 (35)	2 (22.2)	.72 NS	16 (35.6)	5 (35.7)	3 (17.6)	.34 NS	11 (18.3)	12 (75)	*p* < .0001
Male (*n* = 48) *n* (%)	13 (48.1)	28 (70)	6 (66.7)	.19 NS	29 (64.4)	10 (71.4)	8 (47.1)	.33 NS	35 (58.3)	11 (68.8)	.64 NS
Female (*n* = 40) *n* (%)	14 (51.9)	12 (30)	3 (33.3)	.19 NS	16 (35.6)	4 (28.6)	9 (52.9)	.33 NS	25 (41.7)	5 (31.3)	.64 NS

Abbreviations: ICU, intensive care unit; LAC, lactate concentration; M (Q1÷Q3), median and quantiles; NS, not significant.

Observations also showed a statistically significant influence of the administration of catecholamines during the treatment on the ward and patient death.

A total of 81.7% (*n* = 49) of hospitalized patients died in the group of patients treated with catecholamines in the ICU, while in the group wherein treatment did not involve the use of catecholamines, mortality accounted for 18.8% (*n* = 3).

In addition, survival in the whole treatment cycle was higher among the patients in whom catecholamines were not administered.

## 4 DISCUSSION

Our study aimed to assess patients hospitalized in the ICU after IHCA and OHCA using the GOS scale and to assess factors affecting discharge.

A higher rate of survival to hospital discharge was demonstrated by analyzing the data of patients after IHCA from the Danderyd Hospital in Sweden between January 1, 2012 and December 31, 2017. The result of this study was 34%. Other studies in Sweden showed that the survival rate of patients was significantly reduced by 28% in the 70–79 age group, 20% in the 80–89 group, and 14% in the ≥ 90 age group.^29^ In the University Hospital in Cork, 27% survival to hospital discharge after IHCA was reported in 2011.^30^ Sandroni et al., in their analysis, reported a survival rate to hospital discharge in various hospitals after IHCA that ranged from 0% to 42%, with the most common rate being approximately 20%, which is lesser than the value found in our results (27%).^31^

In our center, the rate of survival to hospital discharge for patients after OHCA in 2016 was 34%, which was slightly lower than that in 2015 (37%).^28^

However, this is a much higher result than the one described in England in 2014, which was 7.9%^38^ and in the patients after OHCA in Andalusia from January 2008 to December 2012 (11.2%).^25^

Boyce et al.,^32^ in their analysis, including 242 patients with OHCA at the cardiac center in Medical University Center in Leiden in 2011–2013, reported survival to hospital discharge in 105 patients (43%). The above‐mentioned surprisingly high survival rate accompanied by the mean patient age of 64.8 years (higher than in the current analysis) was probably due to the profile of the hospital and round‐the‐clock availability of the specialists at the cardiac center.

In the Netherlands, an analysis on patients admitted after OHCA in 2001–2010, at the University Medical Center Groningen, which provides 24/7 emergency care in the region, was conducted.

The studies showed a 51% rate of survival to hospital discharge (57% for the group of patients aged <75 years and 33% for the group of patients aged ≥75 years).^33^

In northern India, from January 2014 to April 2015, the rate of survival to hospital discharge was extremely low at 8.8%, where the survival rate by admission was only 32.5%, which is less than the rate of survival to hospital discharge in our analysis. Most patients in the study in the OHCA group aged between 51 and 60 years, which is in contrast to our results, where patients >65 years represented the largest proportion in this group (48.6%). In terms of sex, similar results were obtained. Most patients in the OHCA group were males.^34^

Comparable observations were made by Gajewski et al.,^35^ who noted survival to hospital discharge at a rate of 9% and ROSC at a rate of 55% in their retrospective analysis of 40 cases of IHCA in 2017 during the first half‐year at UCK in Katowice. Nowińska et al.,^36^ in their analysis on the effectiveness of resuscitation in 2015–2016 at WSK in Bydgoszcz, reported a rate of 54% of ROSC after SCA, with only a 14.6% rate of survival to hospital discharge.

The time of hospitalization in the ICU and after ICU discharge were not significantly different in OHCA and IHCA, where the median of these durations was about a week.

Comparison of our results with results from other centers allowed us to observe significant differences in the rate of survival to discharge from the hospital.

The impact on survival probably involved many factors, including the severity of the patients' condition and cardiac arrest mechanism.

In this analysis, we only included patients who developed ROSC after OHCA and IHCA. Nadolny et al. showed that 36% of patients had ROSC after SCA and survival to hospital admission in Poland. Unfortunately, in Silesia, this percentage was lower (27%). The earlier analysis, which concerned only the Silesian region, presented similar statistics.^5^, ^37^

Sip et al. from Poznan included 33 patients in their study on ROSC after OHCA whereas the OHCA group in our analysis comprised 35 patients, and the study period was the same (12 months). Survival to discharge from the hospital in our study was 12 (34.3%) in the OHCA group and 15 (42.7%) in Poznan. However, only five (13.9%) subjects after OHCA showed good neurological status in their patients, as opposed to 10 (28.6%) patients in our study.^38^

Studies from the Świętokrzyskie Province indicate that 43.3% of patients survived hospital discharge, which is greater than that in our study (34.3%). These differences may be due to the nature of the hospital; our hospital is a trauma center. Moreover, their analysis does not consider the neurological condition, and as we know, discharge in a good neurological state is as important as discharge from the hospital.^39^

Numerous retrospective analyses were performed to determine factors influencing the survival rate of patients admitted to ICUs after SCA of different mechanisms with ROSC.

In a similar retrospective analysis performed at our center in 2015,^28^ we observed a negative correlation between LAC >8 mM, as well as the need for treatment with catecholamines and the rate of discharge from the ICU; however, the need for treatment with catecholamines did not determine the rate of hospital discharge contrary to LAC.

In the current retrospective analysis, LAC >8 mM influenced neither the rate of discharge from the ICU nor from the hospital. Nevertheless, the need for treatment with catecholamines significantly deteriorated the rate of discharge from the ICU and hospital.

Many studies have shown a positive correlation between LAC and mortality.^40^, ^41^, ^42^ Patients admitted to the ICU in Frankfurt in 2007–2010 had a poor prognosis with LAC >6.94 mmol/L. In addition, the prognosis in patients with a pH <7.21 was poor.^43^ The current study, as well as the recent study,^28^ did not show a significant correlation between pH and ICU discharge or hospital discharge.

A quarter of all patients were treated with catecholamines after SCA. Over 80% of patients not treated with catecholamines (*n* = 16) survived ICU discharge, and 75% of this group survived until discharge from the hospital. Only 18.3% of subjects undergoing treatment with catecholamines (*n* = 60) survived until discharge. In the research^28^ conducted at our hospital in 2015, treatment with catecholamines had a significant influence only on ICU discharge.

Catecholamine infusion in connection with high LAC within the first 24 h significantly increased the mortality rate, as proven by Donnino et al. in their research during 2011–2012.^43^

In the study by Seeger et al.,^41^ the mean patient age was 66 years (66% males), whereas the survival rate with a good neurological condition accounted for 33.5%.

An analysis performed in Andalusia on a group of patients after OHCA showed that 10.2% of patients were discharged from the hospital with a good neurological outcome defined as CPC 1–2.^32^

Donnino et al.,^43^ in their retrospective multicenter analysis concerning patients with the incidence of OHCA with ROSC in the year 2011–2012, observed a survival rate of 46%, including 30% in good neurological condition. In 97% of the cases, hypothermic therapy was applied, which could have a marked positive influence on the survival rate.

According to our current analysis, the treatment time in the ICU is significantly longer in patients with a poor neurological state (GOS 2–3) than in patients who are in a better condition (GOS 4–5). In our previous study, the results were similar.^28^ In addition, the median duration of hospitalization in the ICU in the GOS 1 group was the same in 2015 and 2016 at our center. Researchers who analyzed the ICU patient discharge based on the analysis of the Silesian ICU Registry noted similar results as did the 2015 and 2016 studies conducted at our hospital.^44^

Patient mortality was lower in 2015 than in 2016 (57% vs. 68%); however, more patients were discharged from the ICU in a bad neurological condition (20% in 2015 and 8% in 2016). These results suggest that more patients died due to treatment complications, such as pneumonia in 2016, and what is worth noting at this point is the lack of appropriate regulations for discharge from ICU to care and curative institutions in Poland.

However, the hospital profile (trauma center), groups with fewer patients, and different etiologies of SCA can influence the results.

The main limitation of the current study is that some patients with a poor neurological condition who were admitted to the ICU after SCA with ROSC died in the ICU because of prolonged treatment complications.^28^

## 5 CONCLUSIONS

In summary, in the analyzed patient groups, treatment in the ICU in 2016, the survival to ICU discharge after SCA with ROSC averaged 32.1% to ICU discharge and 30.8% to hospital discharge. A total of 24.4% of patients survived with good health conditions. A significant statistical difference was not observed in ICU and hospital discharge independently depending on the site of SCA (OHCA vs. IHCA) or SCA mechanism (cardiogenic vs. non‐cardiogenic). We did not observe a statistically significant difference between the health condition and the mechanism or site of SCA. The need for treatment with catecholamines was correlated with increased mortality (GOS 1). Patients with severe conditions had a prolonged duration of hospitalization in the ICU compared with patients having good health conditions (GOS 4–5).

## CONFLICT OF INTEREST

The authors declare no conflict of interests.

## AUTHOR CONTRIBUTION

Michał Stasiowski conceived the concept of the study and supervision. Michał Stasiowski, Jakub Gąsiorek, and Przemysław Jałowiecki contributed to the design of the research. All authors were involved in data collection. Dominika Majer, Ewa Niewiadomska, and Seweryn Król analyzed the data. Jakub Żak, Anna Missir, Lech Krawczyk writing the original‐draft. Beniamin Oskar Grabarek contributed to the formal analysis. All authors edited and approved the final version of the manuscript.

## Data Availability

The data used to support the findings of this study are included within the article.
